# Optimization of Adjuvant Radiation in Breast Conservation Therapy: Can We Minimize without Compromise?

**DOI:** 10.4061/2011/321304

**Published:** 2011-10-05

**Authors:** Sophia M. Edwards-Bennett, Candace R. Correa, Eleanor E. Harris

**Affiliations:** Department of Radiation Oncology, Moffitt Cancer Center, 12902 Magnolia Drive, Tampa, FL 33612, USA

## Abstract

Adjuvant breast radiation therapy after breast conservation surgery is recommended as it yields significant reduction in the risk of local recurrence, and confers a potential overall survival benefit. Although the standard breast radiation regimen has historically been delivered over 5–7 weeks; more novel, shorter courses of breast radiation are currently being employed, offering the advantage of more convenience and less time-commitment. Herein, we review the recent literature substantiating these abbreviated radiation treatment approaches and the methods of delivery thereof. In addition, we discuss imaged guided techniques currently being utilized to further refine the delivery of adjuvant breast radiation therapy.

## 1. Introduction

Multiple randomized studies have demonstrated equivalent survival outcomes with mastectomy versus breast conservation therapy (BCT, breast conservation surgery (BCS), and adjuvant radiation therapy) in the treatment of early stage breast cancer [1, 2]. In addition, the Oxford meta-analysis convincingly demonstrated not only a significant local control benefit but also an overall survival benefit with adjuvant breast radiation therapy after BCS [3]. As such, BCT has been established as the standard of care for limited stage breast cancer offering the advantage of breast preservation, improved quality of life, and cosmesis. Breast conservation surgery, except in rare cases, is followed by adjuvant radiation therapy (RT). A typical adjuvant radiation course is 45 to 50 Gy in 25 to 28 fractions (1.8 to 2 Gy per fraction) delivered to the whole affected breast. A boost of 10–16 Gy in 5 to 8 fractions is usually prescribed to the lumpectomy cavity for additional local control benefit as demonstrated by two seminal studies [4, 5]. 

With the standard RT schedule, a 5–7 weeks commitment is required. For most patients, this is quite inconvenient and cumbersome because of employment or social responsibilities, often confounded by remote distance from the treatment center to their place of work or residence. In fact, this treatment time commitment has been cited as one of the main reasons for noncompliance with adjuvant breast radiation [6]. This demand served as impetus for the development of abbreviated regimes for whole breast radiation. 

One such schedule is the widely adopted Canadian fractionation, 42.5 Gy in 16 fractions (2.65 Gy per fraction) given over 3 weeks [7]. This and other regimens, 40 Gy in 15 fractions and 39 Gy in 13 fractions delivered over 3 weeks, partly borne from its inception, are discussed in detail herein [7–11]. 

Conceptually, a shorter course of radiation therapy necessitates hypofractionation utilizing a higher dose per fraction to achieve radiobiological equivalent effectiveness of the standard, more protracted schedule [12]. From radiobiological principles, since late reacting normal tissues are more sensitive to increasing dose per fraction, a priori, larger dose per fraction should yield more long-term toxicity [12]. Thus, the tenable, reserved position among some physicians is that the abridged regimens may be “tagged” with the high clinical price of long-term treatment toxicities. Thus the lingering question is can we minimize treatment time without compromising toxicity?

The utilization of hypofractionated whole breast RT has been contemporaneous with a more focused approach, accelerated partial breast radiation therapy (APBI). As the name implies, radiation is targeted to the partial breast only, defined by the lumpectomy cavity borders and up to a 2 cm margin diametrically [13]. This regime employs several different treatment methods to deliver an accelerated hypofractionated course of radiation, with a schedule ranging from 20 Gy administered in 1 fraction as in the case of intraoperative radiation therapy (IORT) to 38.5 Gy in 3.85 Gy fractions twice daily over 5 consecutive days with external beam radiation. 

 The underlying principle of partial breast irradiation is that over 85% of all ipsilateral breast recurrences occur in the same quadrant within a 1-2 cm radius of the index lesion. By this premise, partial breast RT should not significantly compromise treatment outcomes compared to whole breast radiation, in low-risk patients [13]. In addition, organs at risk (OAR) for radiation-induced toxicity such as the unaffected contralateral breast, lungs and heart should be less threatened by partial breast than with whole breast radiation therapy.

Partial breast radiation offers a clinically desirable constellation of advantages over conventional radiation, including shortened treatment duration and reduction of normal tissue toxicity. However, this approach inherently assumes accurate definition of the lumpectomy cavity. This begs the question: can we confidently* minimize* target volume *without compromising* treatment outcomes? Image guidance promises to provide improved accuracy in target localization that should allow target volume reduction.

Herein, we review the results from the most relevant hypofractionated whole and partial breast studies and discuss the implications thereof. We conclude with a brief discussion of image guidance and its utility in whole and partial breast radiation.

## 2. Hypofractionated Whole Breast Irradiation

Several randomized clinical trials have compared the efficacy of whole breast radiotherapy with conventional fractionation (i.e., 1.8–2.0 Gy fractions) requiring five to six weeks of daily treatments versus hypofractionated (i.e., >2 Gy fractions) radiotherapy requiring fewer treatments. Overall, these trials have shown equivalent local control of breast cancer and breast cosmesis with conventionally fractionated versus hypofractionated regimens. A Canadian trial compared hypofractionated whole breast regimen delivering a dose of 42.5 Gy in 16 fractions of 2.66 Gy daily fractions over 22 days to conventional fractionation of 50 Gy in 25 daily fractions of 2 Gy each [14]. Both regimens were prescribed without a sequential lumpectomy cavity boost. With median followup of 12 years, the 10-year local control was 93.3% versus 93.8% (*P* > 0.05) for the conventional versus hypofractionated radiation, respectively, with both regimens yielding equivalent cosmesis. 

Another trial centered at the Royal Marsden Hospital compared conventional 50 Gy in 2 Gy fractions to two hypofractionated arms, delivering 39 Gy or 42.9 Gy in thirteen 3.0 Gy or 3.3 Gy fractions, respectively, over 35 days. That study yielded equivalent 10-year local control rates of 87.9%, 85.2%, and 90.4% for conventionally fractionated and the two hypofractionated radiotherapy regimens, respectively [15]. 

Two additional UK trials, START A and START B (Standardization of Breast Radiotherapy), with shorter followup (5-year) similarly demonstrated equivalent local control between treatment arms [16, 17]. 

In contrast, an ongoing UK FAST trial (Faster Radiotherapy for Breast Cancer Radiotherapy) randomized patients between conventionally fractionated radiotherapy and two hypofractionated schedules, 28.5 Gy in five 5.7 Gy fractions and 30 Gy in five 6 Gy fractions delivered over 35 days. The results of this trial have not yet been reported in full-text form [18, 19].

The question of feasibility of delivering lumpectomy cavity boost after Canadian and other fractionated whole breast schedules has been posed. Both START and the Royal Marsden trials prescribed a boost in more than 30% of the study cohort [15–17]. This has also been addressed by a single institution Memorial Sloan Kettering Cancer Center retrospective series in which hypofractionated whole breast radiation therapy (42.4 Gy in 16 fractions of 2.65 Gy each) was delivered to 128 patients followed by a conventionally fractionated boost of (10 Gy in 5 fractions of 2 Gy each) [20]. That study showed comparable cosmetic outcomes to conventional fraction, and there were no grade 3 or more toxicities recorded after median followup of 1.5 years. Another large single institution UK series confirmed feasibility and favorable outcomes with Canadian fraction followed by boost [21].

In 2010, the American Society for Radiation Oncology (ASTRO) published evidence-based guidelines for hypofractionated whole breast radiotherapy [22]. Suitable candidates for hypofractionated radiotherapy are identified as women aged ≥50 years with pT1-2 N0 M0 tumors and who do not receive cytotoxic chemotherapy. With the latter criterion, there is the advantage of less delay in the delivery of radiotherapy. It is still uncertain whether all women benefit equally from hypofractionated as compared to conventionally fractionated radiotherapy regimens. A retrospective exploratory subgroup analysis of the Canadian trial revealed that the hypofractionated regimen was less effective among women with high-grade tumors (10-year local recurrence 15.6% versus 4.7%, hypofractionated versus conventional fractionation regimens, resp.) [14]. Additional data and continued followup from randomized trials will be important in determining the long-term efficacy and cosmesis from hypofractionated whole breast radiotherapy regimens.

## 3. Accelerated Partial Breast Irradiation

Accelerated partial breast irradiation (APBI) is another technique used to deliver a course of radiotherapy over an even shorter time frame of, usually, 5 days. This regimen is offered to a select subset of patients [23]. APBI targets the tissue in the periphery of the lumpectomy tumor bed only. This volume can be targeted with various radiotherapy techniques such as external beam radiotherapy (either 3-D conformal, intensity modulated, or electron radiation therapy), brachytherapy (interstitial or balloon catheter), and intraoperative radiotherapy (electrons or superficial photons). Several randomized clinical trials comparing whole breast to accelerated partial breast irradiation are ongoing. The NSABP B-39/RTOG 0413 trial, goal accrual of 4300, randomizes patients between whole breast irradiation or accelerated partial breast irradiation (with choice of either high-dose rate interstitial brachytherapy, MammoSite balloon catheter, or 3D conformal external bream radiotherapy technique) to a dose of 34 Gy or 38.5 Gy in 3.4 Gy or 3.85 Gy fractions over 5–10 days. Final results of this trial have not yet been reported.

The largest reported APBI trial to date, TARGIT (Targeted Intraoperative Radiotherapy) trial, randomized 2232 women (excluding patients with certain high-risk clinicopathologic features) between whole breast irradiation and a single dose of 20 Gy with intraoperative radiotherapy with superficial low-energy photons. At 4 years, there was no difference in local control between the whole breast (99.1%) and partial breast (98.8%) arms [24].

Results employing Electron Intraoperative Therapy (ELIOT) in 1822 women with early stage breast cancer have been published, demonstrating 97.7% local recurrence rate at 3 years and a 5- and ten-year survivals of 97.4 and 89.7%, respectively, while offering reduction of normal tissues to radiation exposure [25].

GEC-ESTRO (Groupe Européen de Curiethérapie—European Society for Therapeutic Radiology and Oncology); RAPID (Randomized Trial of Accelerated Partial Breast Irradiation); IMPORT LOW (Intensity Modulated and Partial Organ Radiotherapy) are examples of current randomized trials evaluating accelerated partial breast radiotherapy versus whole breast irradiation in the treatment of low-risk breast cancer.

 The treatment and cosmetic outcomes of mature and ongoing clinical trials should help to clarify treatment criteria and appropriately stratify patients to partial breast versus whole breast irradiation.

## 4. External Beam Radiotherapy for Breast Cancer Using Image Guidance

Image guided radiotherapy (IGRT) involves the use of localization techniques at the time of daily treatment to verify accurate positioning. The goal of this endeavor is to reduce patient setup variation, in order to facilitate the use of smaller margins around target volumes to be used. Smaller margins should translate into significantly smaller volume of normal tissue radiated which should in turn reduce acute and late normal tissue toxicity. 

In the case of breast cancer treatment, this approach may reduce late effects to the breast tissue, heart, and lungs. Used in conjunction with localization of the target volume, particularly in the case of partial breast irradiation, IGRT may additively improve daily target dose coverage and therefore improve local control outcomes as well. Even in the setting of whole breast treatment, the use of IGRT may facilitate smaller margins as well as advanced techniques such as simultaneous integrated boost and intensity-modulated radiation therapy (IMRT), both of which require a much higher degree of setup certainty to be effectively used in treatment [26, 27].

Aligning to bony anatomy, as is done in MV and KV or cone beam CT imaging, improves setup accuracy compared to the traditionally employed surface tattoos [28, 29]. Using either breast surface or location of intraparenchymal surgical clips improves localization over strictly bony alignment, even when using CT guidance [30]. Still, there is some uncertainty associated with the delineation of the tumor bed target volume because of significant interobserver variability [31, 32]. For example, the Radiation Therapy Oncology Group (RTOG) conducted a multi-institutional interobserver study among nine radiation oncologists specializing in breast cancer, to determine the degree of variability in target volume and organs at risk delineation among three sample cases [33]. They found structure mean overlap of only 72% for the lumpectomy cavity in one case and poor agreement on nodal structures, with percent overlap as low as 10% among different observers [33]. This variation resulted in substantial variations in treatment planning and dose coverage. Consequently, the RTOG has published an atlas of breast, chest wall, nodal regions, and organs at risk to guide a more consistent reproducible approach to contouring [34].

We have recently reported our experience at Moffitt Cancer Center using fiducial-based IGRT in prospective cohort of both whole breast and partial breast patients. We used textured gold fiducial markers, which adheres to the surrounding soft tissue, increasing the likelihood of fiducial stability and consistent visualization. In fact, 100% fiducial visualization on MV imaging and minimal variation, we were able to verify fiducial migration [35]. 

In the partial breast cohort, there was minimal motion due to intrafraction motion from respiration or changes in respiratory motion between 4D CT scans at the time of simulation to the end of treatment [35]. The mean change in distance between fiducials inter- and intrafraction had a small range 2 to 3 mm, well within the range of error of the total size of the fiducial. Fiducial markers position was stable during treatment with no evidence of substantial fiducial migration within a 5 mm range. The position of the center of fiducial mass relative to the center of the seroma was also stable, confirming the stability and applicability and textured fiducials in IGRT in the setting of APBI [35].

Similarly, in the whole breast cohort, small ranges in inter- and intrafraction motion, respiratory motion, and fiducial migration were observed [36]. Our data suggest that, with fiducial-based image guidance, the PTV margin may be safely reduced from the more standard 10 mm to about 5 mm, substantially reducing the volume of normal tissue irradiated unnecessarily. Other investigators using surgical clips or fiducials for breast cancer radiotherapy have reported similar results [37–41].

## 5. Newer Techniques for Breast IGRT

Although online cone beam CT (CBCT) allows much more accurate and reproducible alignment in 3 dimensions to the bony anatomy as compared to surface tattoos or port films alignment, pretreatment CBCT does not guarantee accurate intrafraction delivery [42, 43]. Prolonged treatment times and couch rotation can significantly reduce treatment delivery accuracy [44]. To address this confounder, patient surface setup systems and real time tracking systems have been recently explored. Data suggest that surface imaging may offer more precise setup than laser or tattoo with a similar reduction in error as fiducial-based IGRT [45]. Ultrasound systems for localization and tracking of the tumor bed for daily treatment have also been investigated and have shown good correlation between the position of the tumor bed on 3D ultrasound and CT, forecasting the utility of 3D US in the near future [46]. Similarly, implantable electromagnetic transponder fiducials have been used to track breast tumor bed motion in real time [47]. With the rising concern of cumulative radiation doses with multiple CT imaging and the lifetime risk of secondary cancers, modalities such as 3D ultrasound image guidance may offer an attractive alternative.

## 6. Conclusions

Hypofractionated breast radiation therapy offers the attractive alternative of shorter treatment course which is not only convenient for patients, but also time and cost-effective. The overarching question remains, can we minimize without compromise? Results of randomized and large single-institution studies seem to support the edict that attaining this desirable balance is indeed possible. 

Reducing target volume, as in the case of external beam APBI techniques, calls for more refinement of treatment delivery with image-guided radiation therapy to ensure accurate delivery of high-dose radiation while sparing normal tissue such as heart and lungs.

Various methods of performing IGRT, including implanted fiducials, CBCT, surface mapping, and ultrasound afford measurable improvement in setup error allowing for PTV margin reductions to as much as 5 mm. Each institution should apply the optimal IGRT technology for their clinical practice commensurate with the center's equipment availability, physician, and technician experience. For, whole breast radiation, wherein a larger volume of heart and lung is irradiated, there is a compelling argument to incorporate IGRT in our daily set up, to achieve optimal results with minimal toxicity. For accelerated partial breast irradiation in particular, IGRT should be systematically incorporated into our daily treatment algorithm.

## References

[1] Fisher B, Anderson S, Bryant J (2002). Twenty-year follow-up of a randomized trial comparing total mastectomy, lumpectomy, and lumpectomy plus irradiation for the treatment of invasive breast cancer. *The New England Journal of Medicine*.

[2] Fisher B, Anderson S, Redmond CK, Wolmark N, Wickerham DL, Cronin WM (1995). Reanalysis and results after 12 years of follow-up in a randomized clinical trial comparing total mastectomy with lumpectomy with or without irradiation in the treatment of breast cancer. *The New England Journal of Medicine*.

[3] Clarke M, Collins R, Darby S (2005). Effects of radiotherapy and of differences in the extent of surgery for early breast cancer on local recurrence and 15-year survival: an overview of the randomised trials. *The Lancet*.

[4] Bartelink H, Horiot JC, Poortmans PM (2007). Impact of a higher radiation dose on local control and survival in breast-conserving therapy of early breast cancer: 10-Year results of the randomized boost versus no boost EORTC 22881-10882 trial. *Journal of Clinical Oncology*.

[5] Romestaing P, Lehingue Y, Carrie C (1997). Role of a 10-Gy boost in the conservative treatment of early breast cancer: results of a randomized clinical trial in Lyon, France. *Journal of Clinical Oncology*.

[6] Albain KS, Green SR, Lichter AS (1996). Influence of patient characteristics, socioeconomic factors, geography, and systemic risk on the use of breast-sparing treatment in women enrolled in adjuvant breast cancer studies: an analysis of two intergroup trials. *Journal of Clinical Oncology*.

[7] Whelan T, MacKenzie R, Julian J (2002). Randomized trial of breast irradiation schedules after lumpectomy for women with lymph node-negative breast cancer. *Journal of the National Cancer Institute*.

[8] Yarnold J, Haviland J (2010). Pushing the limits of hypofractionation for adjuvant whole breast radiotherapy. *Breast*.

[9] Owen JR, Ashton A, Bliss JM (2006). Effect of radiotherapy fraction size on tumour control in patients with early-stage breast cancer after local tumour excision: long-term results of a randomised trial. *The Lancet Oncology*.

[10] Bentzen SM, Agrawal RK, Aird EG (2008). The UK Standardisation of Breast Radiotherapy (START) trial A of radiotherapy hypofractionation for treatment of early breast cancer: a randomised trial. *The Lancet Oncology*.

[11] Bentzen SM, Agrawal RK, Aird EG (2008). The UK Standardisation of Breast Radiotherapy (START) Trial B of radiotherapy hypofractionation for treatment of early breast cancer: a randomised trial. *The Lancet*.

[12] Williams MV, Denekamp J, Fowler JF (1985). A review of *α*/*β* ratios for experimental tumors: implications for clinical studies of altered fractionation. *International Journal of Radiation Oncology Biology Physics*.

[13] Mannino M, Yarnold J (2009). Accelerated partial breast irradiation trials: diversity in rationale and design. *Radiotherapy and Oncology*.

[14] Whelan TJ, Pignol JP, Levine MN (2010). Long-term results of hypofractionated radiation therapy for breast cancer. *The New England Journal of Medicine*.

[15] Yarnold J, Ashton A, Bliss J (2005). Fractionation sensitivity and dose response of late adverse effects in the breast after radiotherapy for early breast cancer: long-term results of a randomised trial. *Radiotherapy and Oncology*.

[16] Bentzen SM, Agrawal RK, Aird EG (2008). The UK Standardisation of Breast Radiotherapy (START) Trial A of radiotherapy hypofractionation for treatment of early breast cancer: a randomised trial. *The Lancet Oncology*.

[17] Bentzen SM, Agrawal RK, Aird EG (2008). The UK Standardisation of Breast Radiotherapy (START) Trial B of radiotherapy hypofractionation for treatment of early breast cancer: a randomised trial. *The Lancet*.

[18] Yarnold J, Bloomeld D, LeVay J (2004). Prospective randomized trial testing 5.7 Gy and 6.0 Gy fractions of whole breast radiotherapy in women with early breast cancer (FAST) trial. *Journal of Clinical Oncology*.

[19] Brunt AM, Sydenham M, Bliss J (2009). A 5-fraction regimen of adjuvant radiotherapy for women with early breast cancer: first analysis of the randomised UK FAST trial. *European Journal of Cancer*.

[20] Croog VJ, Wu AJ, McCormick B, Beal KP (2009). Accelerated whole breast irradiation with intensity-modulated radiotherapy to the prone breast. *International Journal of Radiation Oncology Biology Physics*.

[21] Plataniotis GA, Theofanopoulou MA, Sotiriadou K, Kyrgias G (2010). Hypofractionated radiotherapy for breast cancer patients treated by breast-conserving surgery: short-term morbidity and preliminary results. *Breast Cancer*.

[22] Smith BD, Bentzen SM, Correa CR (2011). Fractionation for whole breast irradiation: an American Society for Radiation Oncology (ASTRO) Evidence-Based Guideline. *International Journal of Radiation Oncology Biology Physics*.

[23] Smith BD, Arthur DW, Buchholz TA (2009). Accelerated partial breast irradiation consensus statement from the American Society for Radiation Oncology (ASTRO). *International Journal of Radiation Oncology Biology Physics*.

[24] Vaidya JS, Joseph DJ, Tobias JS (2010). Targeted intraoperative radiotherapy versus whole breast radiotherapy for breast cancer (TARGIT-A trial): an international, prospective, randomised, non-inferiority phase 3 trial. *The Lancet*.

[25] Veronesi U, Orecchia R, Luini A (2010). Intraoperative radiotherapy during breast conserving surgery: a study on 1,822 cases treated with electrons. *Breast Cancer Research and Treatment*.

[26] Hector CL, Webb S, Evans PM (2000). The dosimetric consequences of inter-fractional patient movement on conventional and intensity-modulated breast radiotherapy treatments. *Radiotherapy and Oncology*.

[27] Frazier RC, Vicini FA, Sharpe MB (2004). Impact of breathing motion on whole breast radiotherapy: a dosimetric analysis using active breathing control. *International Journal of Radiation Oncology Biology Physics*.

[28] Yue NJ, Goyal S, Zhou J, Khan AJ, Haffty BG (2010). Intrafractional target motions and uncertainties of treatment setup reference systems in accelerated partial breast irradiation. *International Journal of Radiation Oncology, Biology, Physics*.

[29] Smith RP, Bloch P, Harris EE (2005). Analysis of interfraction and intrafraction variation during tangential breast irradiation with an electronic portal imaging device. *International Journal of Radiation Oncology Biology Physics*.

[30] Hasan Y, Kim L, Martinez A, Vicini F, Yan D (2008). Image guidance in external beam accelerated partial breast irradiation: comparison of surrogates for the lumpectomy cavity. *International Journal of Radiation Oncology Biology Physics*.

[31] Struikmans H, Wárlám-Rodenhuis C, Stam T (2005). Interobserver variability of clinical target volume delineation of glandular breast tissue and of boost volume in tangential breast irradiation. *Radiotherapy and Oncology*.

[32] Goldberg H, Prosnitz RG, Olson JA, Marks LB (2005). Definition of postlumpectomy tumor bed for radiotherapy boost field planning: CT versus surgical clips. *International Journal of Radiation Oncology Biology Physics*.

[33] Li XA, Tai A, Arthur DW (2009). Variability of target and normal structure delineation for breast cancer radiotherapy: an RTOG multi-institutional and multiobserver study. *International Journal of Radiation Oncology Biology Physics*.

[35] Park CK, Zhang G, Forster KM, Harris EER (2009). Evaluation of fiducial marker migration and respiratory-induced motion for image guided radiotherapy in accelerated partial breast irradiation. *International Journal of Radiation Oncology Biology Physics*.

[36] Latifi K, Forster KM, Harris EER (2009). Evaluation of fiducial marker migration and respiratory-induced motion for image guided radiotherapy in whole breast irradiation. 32nd Annual San Antonio Breast Cancer Symposium. *Cancer Research*.

[37] Dzhugashvili M, Pichenot C, Dunant A (2010). Surgical clips assist in the visualization of the lumpectomy cavity in three-dimensional conformal accelerated partial-breast irradiation. *International Journal of Radiation Oncology Biology Physics*.

[38] Weed DW, Yan D, Martinez AA, Vicini FA, Wilkinson TJ, Wong J (2004). The validity of surgical clips as a radiographic surrogate for the lumpectomy cavity in image-guided accelerated partial breast irradiation. *International Journal of Radiation Oncology Biology Physics*.

[39] Kim LH, Wong J, Yan D (2007). On-line localization of the lumpectomy cavity using surgical clips. *International Journal of Radiation Oncology Biology Physics*.

[40] Gierga DP, Riboldi M, Turcotte JC (2008). Comparison of target registration errors for multiple image-guided techniques in accelerated partial breast irradiation. *International Journal of Radiation Oncology Biology Physics*.

[41] Leonard CE, Tallhamer M, Johnson T (2010). Clinical experience with image-guided radiotherapy in an accelerated partial breast intensity-modulated radiotherapy protocol. *International Journal of Radiation Oncology Biology Physics*.

[42] Topolnjak R, Sonke JJ, Nijkamp J (2010). Breast patient setup error assessment: comparison of electronic portal image devices and cone-beam computed tomography matching results. *International Journal of Radiation Oncology Biology Physics*.

[43] White EA, Cho J, Vallis KA (2007). Cone beam computed tomography guidance for setup of patients receiving accelerated partial breast irradiation. *International Journal of Radiation Oncology Biology Physics*.

[44] Cai G, Hu WG, Chen JY (2010). Impact of residual and intrafractional errors on strategy of correction for image-guided accelerated partial breast irradiation. *Radiation Oncology*.

[45] Bert C, Metheany KG, Doppke K, Chen GTY (2005). A phantom evaluation of a stereo-vision surface imaging system for radiotherapy patient setup. *Medical Physics*.

[46] Wong P, Muanza T, Reynard E, Robert K, Barker J, Sultanem K (2011). Use of three-dimensional ultrasound in the detection of breast tumor bed displacement during radiotherapy. *International Journal of Radiation Oncology Biology Physics*.

[47] Afghan MKN, Eulau S, Morris A (Abstract 10945, AAPM, 2009). *Initial Clinical Experience with Electromagnetic Localization and Tracking for External Beam Partial Breast Irradiation*.

